# Novel Organism Verification and Analysis (NOVA) study: identification of 35 clinical isolates representing potentially novel bacterial taxa using a pipeline based on whole genome sequencing

**DOI:** 10.1186/s12866-023-03163-7

**Published:** 2024-01-05

**Authors:** Veronika Muigg, Helena M.B. Seth-Smith, Kai-Manuel Adam, Maja Weisser, Vladimira Hinić, Annette Blaich, Tim Roloff, Ulrich Heininger, Hanna Schmid, Maurus Kohler, Lukas Graf, Dylan M. Winterflood, Pascal Schlaepfer, Daniel Goldenberger

**Affiliations:** 1 Clinical Bacteriology and Mycology, University Hospital Basel and University of Basel, Petersgraben 4, Basel, 4031 Switzerland; 2 Applied Microbiology Research, Department of Biomedicine, University of Basel, Basel, Switzerland; 3 Swiss Institute of Bioinformatics, Basel, Switzerland; 4 Division of Infectious Diseases and Hospital Epidemiology, University Hospital Basel, Basel, Switzerland; 5 Infectious Diseases and Vaccinology, University of Basel Children’s Hospital, Basel, Switzerland; 6 Kantonsspital Baselland (Bruderholz, Liestal, Laufen), Bruderholz, Switzerland; 7 Ear, Nose and Throat Department, University Hospital Basel, Basel, Switzerland; 8 Laboratory Medicine, University Hospital Basel and University of Basel, Basel, Switzerland; 9 Present Address: Institute of Medical Microbiology, University of Zurich, Zurich, Switzerland

**Keywords:** Clinical isolates, Difficult to identify strains, Whole genome sequencing (WGS), Novel bacteria, Clinical significance, Type (strain) genome server (TYGS)

## Abstract

**Background:**

Reliable species identification of cultured isolates is essential in clinical bacteriology. We established a new study algorithm named **NOVA – N**ovel **O**rganism **V**erification and **A**nalysis to systematically analyze bacterial isolates that cannot be characterized by conventional identification procedures MALDI-TOF MS and partial 16 S rRNA gene sequencing using Whole Genome Sequencing (WGS).

**Results:**

We identified a total of 35 bacterial strains that represent potentially novel species. *Corynebacterium* sp. (n = 6) and *Schaalia* sp. (n = 5) were the predominant genera. Two strains each were identified within the genera *Anaerococcus*, *Clostridium*, *Desulfovibrio*, and *Peptoniphilus*, and one new species was detected within *Citrobacter*, *Dermabacter*, *Helcococcus*, *Lancefieldella*, *Neisseria*, *Ochrobactrum (Brucella)*, *Paenibacillus*, *Pantoea*, *Porphyromonas*, *Pseudoclavibacter*, *Pseudomonas*, *Psychrobacter*, *Pusillimonas*, *Rothia*, *Sneathia*, and *Tessaracoccus*. Twenty-seven of 35 strains were isolated from deep tissue specimens or blood cultures. Seven out of 35 isolated strains identified were clinically relevant. In addition, 26 bacterial strains that could only be identified at the species level using WGS analysis, were mainly organisms that have been identified/classified very recently.

**Conclusion:**

Our new algorithm proved to be a powerful tool for detection and identification of novel bacterial organisms. Publicly available clinical and genomic data may help to better understand their clinical and ecological role. Our identification of 35 novel strains, 7 of which appear to be clinically relevant, shows the wide range of undescribed pathogens yet to define.

**Supplementary Information:**

The online version contains supplementary material available at 10.1186/s12866-023-03163-7.

## Background

Species identification is the first and crucial step in the workflow of clinical microbiology as it provides essential guidance regarding treatment [1]. While the vast majority of pathogens isolated in clinical microbiology laboratories belong to well characterized species, a small number of bacterial isolates may not be reliably identified using conventional identification methods due to lack of sufficient reference data or to the presence of a previously uncharacterized organisms. In cases where the rapid Matrix-Assisted Laser Desorption/Ionization Time-of-Flight Mass Spectrometry (MALDI-TOF MS) methods do not provide a clear identification, molecular techniques are often used. The establishment of 16 S rRNA gene sequence analysis has provided a simple and rapid method for species identification in such cases, and has led to the reclassification and renaming of numerous bacterial genera and species [2, 3]. However, in some cases, analysis of the 16 S rRNA gene sequence also fails to distinguish between species. In these cases, whole genome sequencing (WGS) can be used, which offers better resolution at the species level [1, 4].

We have established an algorithm to identify and characterize strains which are not identifiable by standard methods, i.e., MALDI-TOF MS and partial 16 S rRNA gene sequence analysis, using WGS in a systematic approach. The aim of the study is to detect and characterize new bacterial organisms isolated from clinical specimens and to reliably detect difficult to identify strains. In this report, we describe 35 isolates that represent novel bacterial species, 7 of which were clinically relevant, as well as 26 strains (22 species) whose identification in the routine laboratory was problematic. We provide genome sequences of these species to expand the public database for taxonomic and epidemiological purposes, and we additionally present detailed clinical information about the patients and an assessment of the clinical relevance of the isolates to gain clinical and ecological knowledge about the novel bacterial species.

## Methods

**Fig. 1:**
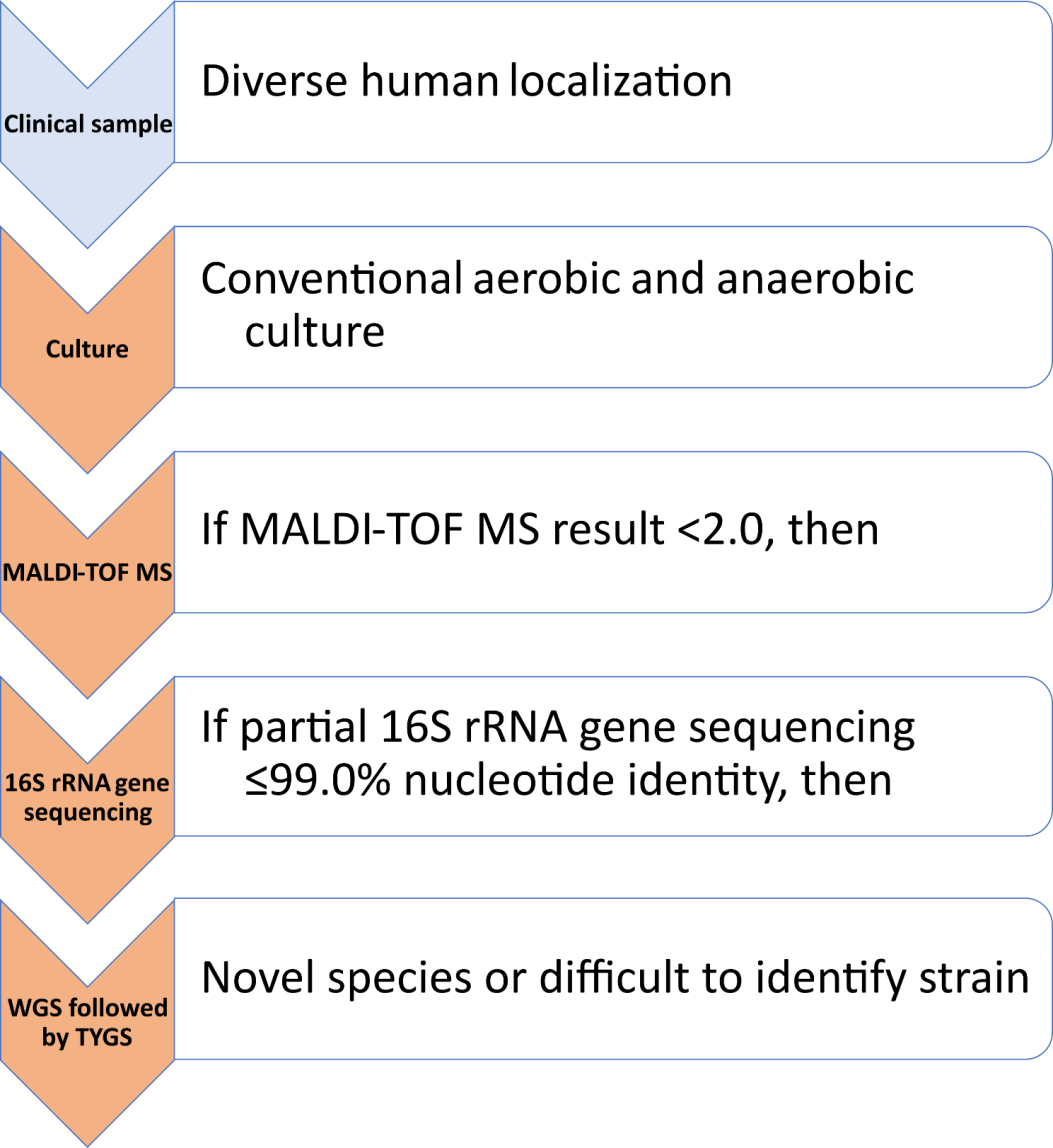
Algorithm for identification of clinical isolates suitable for the NOVA study

The Novel Organism Verification and Analysis (NOVA) study is a prospective study with the aim of characterizing bacterial isolates that are not identifiable by routine diagnostic methods using WGS and thereby describing potential new species. The study was conducted at the Department of Clinical Bacteriology and Mycology of the University Hospital Basel, a tertiary care hospital in Switzerland, and was initiated in 2014. Here we present phenotypic and molecular data on bacterial isolates as well as clinical information on the patients within a time span from December 2014 to January 2022. Isolates that qualified for the NOVA study were identified using a specific algorithm that was integrated into the routine diagnostic process (Fig. 1).

### Description of the NOVA algorithm

Microscopy, aerobic and anaerobic cultures from the various clinical specimens were performed according to standard microbiological procedures including enrichment culture using thioglycolate medium. Anaerobic cultures were incubated and manipulated in an anaerobic workstation (Whitley A 95, Don Whitley Scientific Ltd., Bingley, UK). Species identification of bacterial isolates from routine culture procedures was conducted by MALDI-TOF MS (Bruker Daltonics GmbH, Bremen, Germany) using a simple smear technique with a 1-µl formic acid overlay and cyano-4-hydroxyinnamic acid (CHCA) matrix solution. Measurements were analyzed with the main spectra library Bruker Daltonics database. If no reliable species identification was achieved with MALDI-TOF MS; i.e., score < 2.0, divergent results on the first and second hit, no validly published species, e.g., *Corynebacterium* lipophilic group F1, or no identification on species level, isolates were subsequently analyzed using partial 16 S rRNA gene PCR and sequence analysis of approximately 800 bp of the first part [5]. The resulting sequences were compared to the 16 S rRNA gene sequence nucleotide databases of the National Center for Biotechnology Information (NCBI) network service (https://blast.ncbi.nlm.nih.gov). If seven or more mismatches/gaps (corresponding to ≤ 99.0% nucleotide identity) were identified in the analyzed sequence compared to the closest correctly described bacterial species, the isolates were included into the NOVA study (Fig. 1). A species was considered correctly described if it was designated as validly published in the List of Prokaryotic names with Standing in Nomenclature (LPSN) of the German strain collection database (https://www.bacterio.net) [6].

### The NOVA pipeline

DNA Extraction was executed with EZ1 DNA Tissue Kit using EZ1 Advanced Instrument (Qiagen, Hilden, Germany). WGS was performed using Illumina technology (MiSeq or NextSeq500) following library creation (NexteraXT or Illumina DNA prep). Assemblies were created from trimmed (trimmomatic v 0.38) [7] reads using unicycler v0.3.0b [8] and annotated using Prokka v1.13 [9]. Assemblies were analyzed using rMLST [10] and TYGS (https://tygs.dsmz.de using the 70% digital DNA:DNA hybridization (dDDH) cutoff and method 2 [11]. The date of ultimate TYGS analysis was August 8, 2023.

Average Nucleoted Identity (ANI) values were calculated using the OrthoANIu [12]. Calculations were automated using a windows batch file (GitHub: https://github.com/schlaepferp/win_batch_ani).

### Evaluation of clinical relevance by infectious diseases specialists

Patient data were retrospectively extracted from medical records, and the microbiological findings were evaluated individually along with the patient’s clinical presentation by an infectious disease specialist. Clinical relevance was estimated on the basis of the following criteria: clinical signs and symptoms, presence of concomitant pathogens, pathogenic potential of the genus of the isolate, and clinical plausibility. The impact on patient care in terms of antibiotic use or antibiotic switching was not investigated in our study.

### Availability of data

Genome data of 56 isolates of this study is accessible at NCBI under BioProject number PRJEB55530. Genome data of *Gulosibacter hominis* strains USB_NOVA_36, USB_NOVA_37, and USB_NOVA_38 are available under CAJGWQ000000000, CAJHCD000000000, and CAJHCF000000000, respectively [13]. The genomes of *Pseudoclavibacter triregionum* (USB_NOVA_49) [14] and *Cutibacterium modestum* (USB_NOVA_51) [15] are accessible under OU365335 and PRJEB41775, respectively. Scripts for calculating ANI-values between two genomes are deposited in GitHub [https://github.com/schlaepferp/win_batch_ani].

## Results

A total of 61 isolates, 41 (67%) Gram positive and 20 (33%) Gram negative strains, were not identifiable using routine methods and were included in the NOVA study within the study period. Thirty-five (57%) organisms were identified to be novel bacterial species and 26 (43%) isolates represented difficult to identify organisms.

**Fig. 2:**
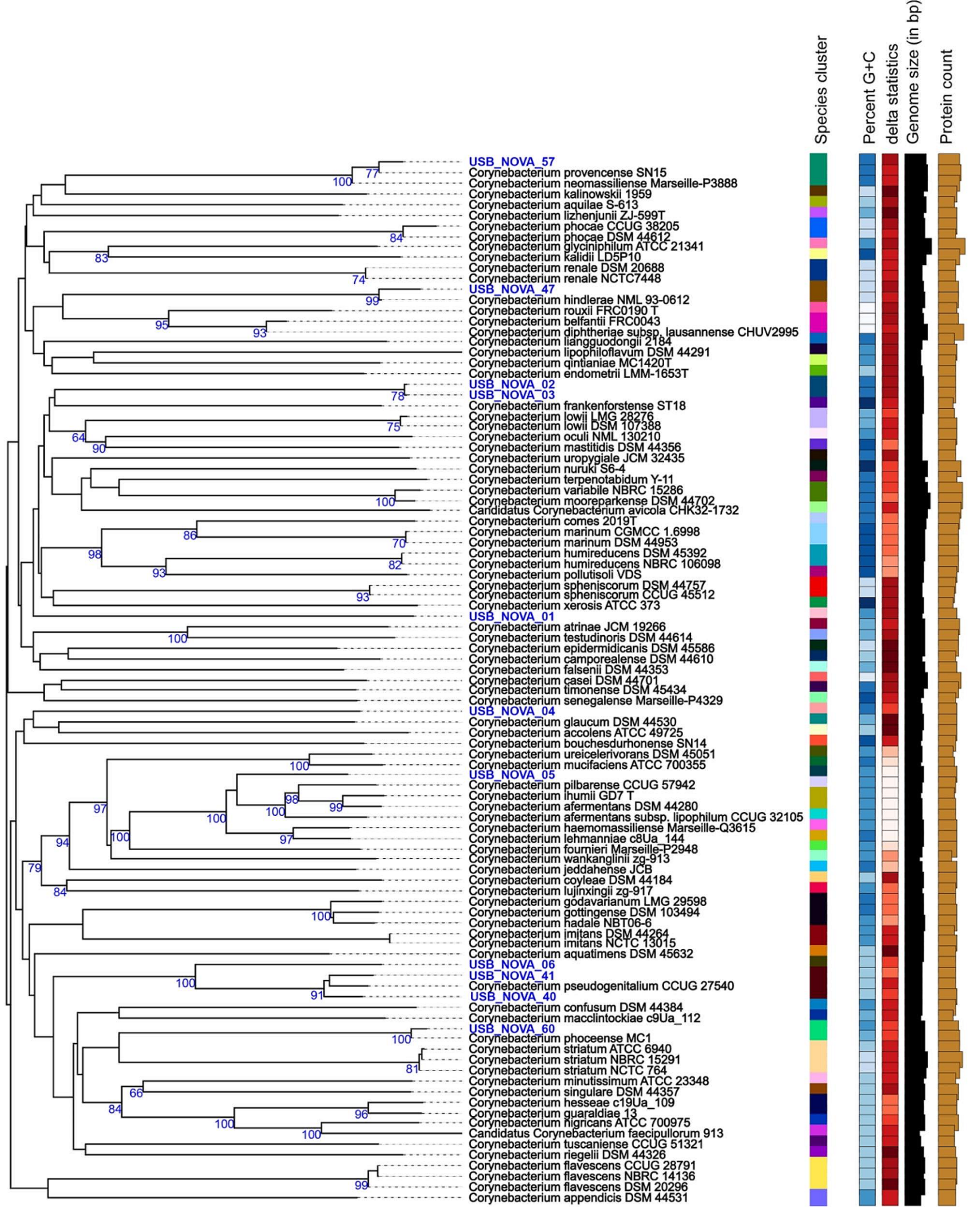
TYGS GBDP tree showing relationships between *Corynebacterium* genomes and references. Figure was generated by TYGS (10.1038/s41467-019-10210-3), with 11 genomes sequenced for this publication shown in blue colour. Species clusters are defined by dDDH with a 70% cutoff shown in the first metadatacolumn. Isolates USB_NOVA_40 and USB_NOVA_41 represent *C. pseudogenitalium*, isolates USB_NOVA_47, USB_NOVA_57, and USB_NOVA_60 cluster with species *C. hindlerae*, “*C. provencense”*, and “*C. phoceense”*, respectively; the other 6 genomes do not form clusters with any sequenced type strains

Predominant genus was *Corynebacterium* with 11 isolates (2 *C. pseudogenitalium*, 1 *C. hindlerae*, 1 “*C. phoceense*”, 1 “*C. provencense*”, and 6 *Corynebacterium* sp. nov. (Fig. 2), followed by *Schaalia* sp. with 5 strains.

The anatomical localization of these 61 clinical samples are indicated in Tables 1 and 2. Predominant specimen was blood culture (n = 9). Detailed microbiological results from the 61 cases including type of specimen, microscopy, cultured isolates, MALDI-TOF MS, and partial 16 S rRNA gene sequencing are listed in Table S1.

**Table 1:** List of 35 clinical isolates representing novel taxa and corresponding clinical data

ID Number	Species	Genbank Accession No. (BioSample): SAMEA	Origin of specimen	Clinical data			
				Age(y)/sex	Clinical presentation	Relevant underlying disease	Clinical relevance
USB_NOVA_01	*Corynebacterium* sp.nov	111,563,050	Swab toe	61/m	Abscess formation	None	Not relevant
USB_NOVA_02	*Corynebacterium* sp.nov	111,563,052	Urine	57/f	Urolithiasis	None	Not relevant
USB_NOVA_03	*Corynebacterium* sp.nov	111,563,049	Swab external auditory canal	53/m	Acute otitis media due to *Pseudomonas aeruginosa*	Squamous cell carcinoma floor of mouth	Not relevant
USB_NOVA_04	*Corynebacterium* sp.nov	111,563,047	Blood culture	55/m	Intoxication with drug of abuse	DM Typ 2	Not relevant
USB_NOVA_05	*Corynebacterium* sp.nov	111,563,048	Blood culture	68/m	Cholangitis	Hepatocellular carcinoma	Not relevant
USB_NOVA_06	*Corynebacterium* sp.nov	111,563,051	Urine	48/m	Urolithiasis	None	Not relevant
USB_NOVA_07	*Schaalia* sp.nov.	111,563,087	Biopsy jaw	90/f	Chronic osteomyelitis of the jaw/ MRONJ	Metastatic breast cancer	Unclear
USB_NOVA_08	*Schaalia* sp.nov.	111,563,089	Swab mouth	65/f	SSI of the mouth and jaw	None	Not relevant
USB_NOVA_09	*Schaalia* sp.nov.	111,563,086	Biopsy submandibular	61/f	Perimandibular abscess	None	Not relevant
USB_NOVA_10	*Schaalia* sp.nov.	111,563,085	Biopsy lung	68/m	Pleural effusion after pleurodesis	Pleuramesothelioma	Unclear
USB_NOVA_11	*Schaalia* sp.nov.	111,563,088	Swab jaw	61/f	Abscess fossa canina	None	Relevant
USB_NOVA_12	*Anaerococcus* sp.nov.	111,563,038	Biopsy bone toe	88/f	Chronic polymicrobial osteomyelitis	Peripheral arterial occlusive disease	Relevant
USB_NOVA_13	*Anaerococcus* sp.nov.	111,563,039	Biopsy bone	93/f	Implant associated infection of the tibia	None	Not relevant
USB_NOVA_14	*Clostridium* sp.nov.	111,563,043	Biopsy hand	38/f	Soft tissue infection	None	Relevant
USB_NOVA_15	*Clostridium* sp.nov.	111,563,042	Blood culture	41/m	Possible pneumococcal pneumonia	IVDU	Unclear
USB_NOVA_16	*Desulfovibrio* sp.nov.	111,563,058	Swab abdomen	64/f	Teritiary peritonitis	Perforation of the rectum	Relevant
USB_NOVA_17	*Desulfovibrio* sp.nov.	111,563,057	Blood culture	N.a.	N.a.	N.a.	N.a.
USB_NOVA_18	*Peptoniphilus* sp.nov.	111,563,074	Biopsy abdomen	N.a.	N.a.	N.a.	N.a.
USB_NOVA_19	*Peptoniphilus* sp.nov	111,563,073	Biopsy upper leg	52/m	Chronic soft tissue inflammation/fistula	DM Typ 2	Unclear
USB_NOVA_20	*Porphyromonas* sp.nov	111,563,075	Abscess mamma	N.a.	N.a.	N.a.	N.a.
USB_NOVA_21	*Pusillimonas* sp.nov	111,563,082	Swab external auditory canal	N.a.	N.a.	N.a.	N.a.
USB_NOVA_22	*Dermabacter* sp.nov	111,563,056	Swab toe	N.a.	N.a.	N.a.	N.a.
USB_NOVA_23	*Helcococcus* sp.nov.	111,563,063	Blood culture	58/m	Spondylodiscitis due to *Streptococcus dysgalactiae*	IVDU	Unclear
USB_NOVA_24	*Neisseria* sp.nov.	111,563,067	Swab leg	71/m	Soft tissue infection	Postoperative wound healing disorder and DM Typ 2	Unclear
USB_NOVA_25	*Pseudomonas* sp.nov	111,563,078	Biopsy hand	67/m	Open fracture	Traumatic amputation of the hand	Relevant
USB_NOVA_26	*Pantoea* sp.nov	111,563,071	Biopsy hand	67/m	Open fracture	Traumatic amputation of the hand	Relevant
USB_NOVA_27	*Lancefieldella* sp.nov	111,563,065	Swab maxilla	67/m	MRONJ	Metastatic prostata carcinoma	Not relevant
USB_NOVA_28	*Rothia sp. nov.*	111,563,083	Swab sacral	26/m	Pilonidal cyst	None	Not relevant
USB_NOVA_29	*Pseudoclavibacter* sp.nov.	111,563,077	Swab external auditory canal	N.a.	N.a.	N.a.	N.a.
USB_NOVA_30	*Tessaracoccus* sp.nov	111,563,092	Biopsy finger	N.a.	N.a.	N.a.	N.a.
USB_NOVA_31	*Citrobacter* sp.nov.	111,563,041	Swab rectal	N.a.	ESBL-screening	Leukemia	N.a.
USB_NOVA_32	*Paenibacillus* sp.nov	111,563,069	Aspirate pleura	42/f	Pulmonary lesion, possible pulmonary fascioliasis	None	Not relevant
USB_NOVA_33	*Ochrobactrum (Brucella)* sp.nov	111,563,040	Bronchial secretion	69/m	Multifactorial respiratory failure, aspiration	Lung cancer, COPD Gold III	Not relevant
USB_NOVA_34	*Sneathia* sp.nov	111,563,091	Swab pharynx	23/m	Retropharyngeal abscess	None	Relevant
USB_NOVA_35	*Psychrobacter* sp.nov.	111,563,081	Blood culture	N.a.	N.a.	N.a.	N.a.

Abbreviations. ID, identification; y, year; f, female; m, male; DM, diabetes mellitus; MRONJ, medication-related osteonecrosis of the jaw;

SSI, surgical site infection; IVDU, intravenous drug use; N.a. not applicable; ESBL, extended spectrum beta-lactamases COPD; chronic obstructive pulmonary disease.

**Table 2:** List of 26 clinical isolates which were identified by using WGS and corresponding clinical data

ID Number	Species / Reference	Genbank Accession No. (BioSample): SAMEA	Origin of specimen	Clinical data				
				Age(y)/sex	Clinical presentation	Relevant underlying disease	Clinical relevance	
USB_NOVA_36	*Gulosibacter hominis*	See ref (13)	Swab external auditory canal	77/m	Otitis media and tympanic drainage	None	Unclear	
USB_NOVA_37	*Gulosibacter hominis*	See ref (13)	Swab external auditory canal	53/m	Acute otitis media	None	Unclear	
USB_NOVA_38	*Gulosibacter hominis*	See ref (13)	Swab external auditory canal	90/m	Chronic otitis media, tympanic membrane perforation	Squamous cell carcinoma of the concha auris, leukemia	Unclear	
USB_NOVA_39	*Gulosibacter hominis*	111,563,062	Biopsy foot	80/f	Implant associated infection due to *Staphylococcus epidermidis* and *Enterococcus faecalis*	None	Not relevant	
USB_NOVA_40	*Corynebacterium pseudogenitalium*	111,563,053	Biopsy placenta fetal site	27/f	Premature vaginal birth	None	Not relevant	
USB_NOVA_41	*Corynebacterium pseudogenitalium*	111,563,055	Urine	60/f	Asymptomatic bacteriuria	Nephrostoma	Not relevant	
USB_NOVA_42	*“Kingella pumchi”*	111,563,068	Swab finger nail	14/m	Paronychia	None	Relevant	
USB_NOVA_43	*Fenollaria massiliensis*	111,563,061	Biopsy bone symphysis	52/m	Possible SSI with fistula of the symphysis pubis	Open-book-fracture of the pelvis four months before	Unclear	
USB_NOVA_44	*Mogibacterium kristiansenii*	111,563,066	Biopsy scrotum	76/m	Fournier gangrene, sepsis due to colo-scrotal fistula	Radiotherapy and resection of the rectum	Relevant	
USB_NOVA_45	*Anaerococcus degeneri*	111,563,037	Biopsy abscess plantar	90/m	Wet gangrene with osteomyelitis calcaneus	Chronic ulcera	Relevant	
USB_NOVA_46	*Slackia exigua*	111,563,090	Biopsy abscess plantar	90/m	Wet gangrene with osteomyelitis calcaneus	Chronic ulcera	Relevant	
USB_NOVA_47	*Corynebacterium hindlerae*	111,563,054	Biopsy bone heel	64/m	Chronic ulcer	None	Not relevant	
USB_NOVA_48	*Devosia equisanguinis*	111,563,059	Blood culture	35/m	Septic thrombophlebitis	IVDU	Relevant	
USB_NOVA_49	*Pseudoclavibacter triregionum*	See ref (14)	Blood culture	7/f	Bacteraemia and fever	Pelvic osteotomy, cerebral palsy,	Unclear	
USB_NOVA_50	*Pseudomonas yangonensis*	111,563,079	Swab lower leg	54/f	Wound healing disorder after compartment syndrome	Hemorrhagic diatheses, liver cirrhosis	Not relevant	
USB_NOVA_51	*Cutibacterium modestum*	See ref (15)	Sonificated fluid prosthetic hip	N.a.	N.a.	N.a.	N.a.	
USB_NOVA_52	*Pseudoramibacter alactolyticus*	111,563,080	Blood culture	86/m	SARS-CoV-2 infection	None	Not relevant	
USB_NOVA_53	*Enterococcus dongliensis*	111,563,060	Aspirate bile	70/f	Cholangitis	Cholangiocellular carcinoma	Relevant	
USB_NOVA_54	*Prevotella brunnea*	111,563,076	Biopsy abdomen	N.a.	N.a.	N.a.	N.a.	
USB_NOVA_55	*Parvimonas parva*	111,563,072	Biopsy tibia	15/m	Osteomyelitis and chronic cutaneous fistula	Osteosarcoma tibia	Relevant	
USB_NOVA_56	*Kingella negevensis*	111,563,064	Swab vaginal	N.a.	N.a.	N.a.	N.a.	
USB_NOVA_57	*“Corynebacterium provencense”*	111,563,046	Urine	87/m	Asymptomatic bacteriuria	Urolithiasis and uretral stent	Not relevant	
USB_NOVA_58	*Vandammella animalimorsus*	111,563,044	Biopsy thumb	38/f	Septic arthritis and tenosynovitis after dog bite	None	Relevant	
USB_NOVA_59	*Saezia sanguinis*	111,563,084	Swab rectal	N.a.	ESBL-screening	None	N.a.	
USB_NOVA_60	*“Corynebacterium phoceense“*	111,563,045	Biopsy back	94/m	Postoperative hematoma/seroma after spine surgery	None	Not relevant	
USB_NOVA_61	*Pantoea agglomerans*	111,563,070	Swab forehead	N.a.	N.a.	N.a.	N.a.	

Abbreviations. ID, identification; y, year; f, female; m, male; SSI, surgical site infection; IVDU, intravenous drug use; N.a. not applicable; ESBL, extended spectrum beta-lactamases

Overall, medical history and information on clinical relevance were available from 47/61 cases. In 15/47 of cases, the respective bacterial isolate was considered clinically relevant, and in 21 cases as not clinically relevant. In the remaining 11 cases, clinical relevance was unclear. In 3/15 cases classified as clinically relevant, culture growth was monomicrobial. In 2 of these 3 cases, patients had received antibiotics for > 3 days at the time of sample collection.

The age range of the 47 patients was from 7 to 94, median age 61 years. Thirty (64%) were males and 17 (36%) females.

### Isolates representing novel species, (n = 35)

Twenty-one isolates grew under aerobic and 14 under anaerobic conditions. Twenty-four (69%) strains were Gram positive and 11 (31%) Gram negative. Six isolates belong to novel species within the genus *Corynebacterium* (Fig. 2), followed by *Schaalia* (n = 5). There were two strains representative for each of the following genera: *Anaerooccus*, *Clostridium*, *Desulfovibrio*, and *Peptoniphilus*. For each of the following genera one new species was identified: *Citrobacter*, *Dermabacter*, *Helcococcus*, *Lancefieldella*, *Neisseria*, *Ochrobactrum (Brucella), Paenibacillus*, *Pantoea*, *Porphyromonas*, *Pseudoclavibacter*, *Pseudomonas*, *Psychrobacter*, *Pusillimonas*, *Rothia*, *Sneathia*, and *Tessaracoccus* (Table 1).

The following isolates represent the same novel species based on an ANI index ≥ 96.0: *Corynebacterium* sp. nov.: isolate USB_NOVA_02 and USB_NOVA_03, ANI 99.96 (Fig. 2); *Desulfovibrio* sp. nov. USB_NOVA_16 and USB_NOVA_17, ANI 98.5; *Peptoniphilus* sp. nov. USB_NOVA_18 und USB_NOVA_19, ANI 97.7.

Clinical data were available from 26 cases. Seven/26, 6/26, and 13/26 were classified clinically relevant, unclear, and clinically not relevant, respectively (Table 1).

### Difficult to identify isolates, (n = 26)

Twenty-six isolates belong to previously described species which could not be identified by standard identification methods, but only by WGS. These strains represent 19 species already validly published and three species not yet validly published. (Table 2). Seventeen (65.4%) strains were Gram-stain-positive and 9 (34.6%) Gram-stain-negative. Four isolates were identified as *Gulosibacter hominis*, and one isolate as *Pseudoclavibacter triregionum.* Both aerobic Gram-stain-positive bacilli have been described taxonomically from our group in collaboration with the BCCM/LMG Bacteria Collection, Ghent, Belgium in 2021 and 2022, respectively [13, 14]. Two isolates represent *Corynebacterium pseudogenitalium* that has been published validly very recently [16]. In addition, one isolate for each of the following species were identified: *Anaerococcus degeneri*, *Corynebacterium hindlerae, Corynebacterium phoceense, Corynebacterium provencense, Cutibacterium modestum*, *Devosia equisanguinis*, *Enterococcus dongliensis*, *Fenollaria massiliensis*, *Kingella negevensis*, *Kingella pumchi, Mogibacter kristiansenii*, *Pantoea agglomerans*, *Parvimonas parva*, *Prevotella brunnea*, *Pseudomonas yangonensis*, *Pseudoramibacter alactolyticus*, *Saezia sanguinis*, *Slackia exigua*, and *Vandamella animalimorsus*. Of these, “*Corynebacterium phoceense”*, “*Corynebacterium provencense”, and “Kingella pumchi”* represent not yet validly published bacterial species. Fifteen (63.6%) from a total of 22 species have been described recently (≥ year 2019) and 14 (53.8%) out of 26 isolates have been isolated before the valid description of the species.

Clinical data were available from 21 cases. Eight/21, 5/21, and 8/21 were classified clinically relevant, unclear, and clinically not relevant, respectively (Table 2).

## Discussion

We present an innovative algorithm based on WGS for systematic and reliable identification of bacterial isolates that can not be identified by routine diagnostic methods. Using this algorithm, we collected and analyzed a total of 61 clinical isolates, 35 of them represent potentially novel species and from February 2022 to July 2023 another 21 potentially novel isolates have been collected (not presented in this publication).

The idea of this study arose with the introduction of the WGS technology in our laboratory. Initially, analysis of the genomes was performed in individual time-consuming procedures. A milestone was the availability of the web-based TYGS platform in 2019, which allows genomic data to be analyzed in a standardized manner to determine the correct taxonomic species or define the organism as a novel taxon based on WGS data [11]. Our NOVA tool is now integrated in routine diagnostic procedures and is performed weekly. It represents a relatively fast and reliable tool to identify difficult to identify bacterial strains and allows to discuss the clinical relevance with our infectious disease specialists in a timely manner.

The predominant genus among our 61 NOVA isolates was *Corynebacterium* with 11 isolates. Five of them were difficult to identify and six represent novel species **(**Fig. 2**)**. Non-diphtheria corynebacteria are part of the normal microbiota of human skin and mucosa and are therefore very common isolates in clinical samples [17]. This may explain our finding, as well as the fact that none of the 11 corynebacteria isolates were considered clinically relevant. However, the growing number of immunocompromised patients and the use of invasive devices are accompanied by an increase in infections with opportunistic pathogens [17, 18]. For this reason and due to the different antibiotic resistance patterns of the different *Corynebacterium* sp., the identification of this bacterial group on species level is of great importance [19]. For this purpose, in addition to MALDI-TOF-MS analysis, various molecular methods such as PCR-based assays or sequencing of the *rpoB* and 16 S rRNA gene have been described [17, 20–23]. However, a recent review by Church and colleagues states that approximately 35% of *Corynebacterium* sp. cannot be distinguished using 16 S rRNA gene sequencing [24]. In these cases, sequencing of the *rpoB* target may provide additional diversity to distinguish some closely related species [21]. WGS, with its higher resolution, ultimately offers another means of species identification as well as the advantage of being able to describe the entire genome of a potentially new species.

We assume, that *Vandammella animalimorsus* represents a novel and emerging pathogen. Our isolate USB_NOVA_58 originated from a biopsy of a thumb after a dog bite with the clinical diagnosis of septic arthritis and tenosynovitis in 2021. It was identified at that time as a potentially novel organism classified as *Corticibacter* sp. After reanalysis using the TYGS tool in 2023, the isolate was now identified as *V. animalimorsus*. This novel genus and species was described by Bernard et al. in 2022 using strains provisionally named “CDC group NO-1” recovered from human wound infections following animal bites [25]. Another potential new pathogen is *Kingella pumchi*. Our strain USB_NOVA_42 was isolated in 2018 from a patient with paronychia and assessed as clinically relevant. At that time it was identified as novel organism tentatively named “unidentified *Neisseria* sp.”. It was described as “*Kingella pumchi”* in February 2023 by a Chinese group using a strain, that had been isolated from a human vertebral biopsy [26]. A novel *Cutibacterium*, *C. modestum*, was identified from a prostethic hip fluid. We identified this strain (USB_NOVA_51) in 2020 as “*Propionibacterium humerusii*”, a tentatively named species published in 2011. Some weeks afterwards, *C. modestum* was described by Dekio I. et al. from an isolate obtained from the meibomian gland [27] showing similar genome data to our strain USB_NOVA_51. We then summarized multiple published data on this organism and showed that “*P. humerusii*” and *C. modestum* represent the same species und that this bacterium often is misidentified as *Cutibacterium acnes* [15]. The recently described *Gulosibacter hominis* (4 isolates) and *Pseudoclavibacter triregionum* (1 isolate) may represent commensals that are part of the human skin microbiome [13, 14].

As a strength of our study, we identified and described novel species from clinical samples, while also providing clinical information and evaluating the clinical relevance of the respective bacterial isolate. In approximately one-third (15/47) of all cases where clinical data were available, the bacterial isolate was considered clinically relevant. However, in 12/15 cases, other concomitant pathogens could be identified as possible cause of the infection, so determination of their clinical relevance was difficult. Moreover, we did not evaluate antibiotic efficacy or change in antibiotic administration based on strain identification. This is a limitation of this study because the impact on patient care is difficult to assess without this information. In 11/47 cases the clinical relevance of the isolate was unclear. Six of these 11 isolates belong to novel species. This demonstrates the importance of identifying bacterial species and collecting clinical data on patients to gain insight into the role of these species as a human pathogen and to better assess their clinical significance in the future.

In our findings, 26 of 61 isolates were difficult to identify at the time point of study inclusion, when combining MALDI-TOF MS testing with partial 16 S rRNA gene sequencing. However, the long collection time limits this classification. Technical advances occurring within the timeframe of study inclusion and reporting may or may not allow for identification with one or both of the methods. Yet our NOVA algorithm was implemented to detect novel species which led to 35 of 61 strains being classified as such at timepoint of reporting (August 8 2023).

Overall, the majority (33/61, 54%) of our isolates were Gram positive rods, which are generally difficult to identify biochemically. This is consistent with observations from other laboratories. Church and colleagues found that the largest group of organisms to be sequenced were Gram-positive bacilli, which accounted for 48.5% of all isolates sequenced over a six-year period [24].

The implementation of WGS in clinical microbiology for pan-bacterial identification seems to be more challenging and this method is currently performed mainly at large reference and public health laboratories [28, 29]. Difficulties arise from the lack of guidelines and standards, as well as financial and technical obstacles [28]. Price and colleagues conducted a study using WGS to identify bacteria in a clinical laboratory, evaluated their clinical relevance, and thereby provided a model for validating and implementing WGS in such a setting. They used a diverse set of 125 bacterial isolates, and were able to identify 100% (89/89) and 89% (79/89) of isolates to genus and species levels, respectively. WGS also provided better results for isolates (71% (25/35) originally reported at the genus level or descriptively only. In addition, review of patient records showed that improved identification at the genus or species level through WGS may have had a positive impact on patient care. For example, unnecessary use or use of ineffective antibiotics could be identified, as well as the results provide assistance with outbreak investigations [28, 29]. These benefits of WGS is weighed against the question of clinical practicability with regard to the long turnaround time (1–2 weeks in Price´s study). Faster WGS methods, such as nanopore sequencing, could help overcome this problem [30].

Current literature indicates that the MALDI-TOF MS method identifies approximately 98% of routine clinical isolates to the genus level and > 90% to the species level, with < 1% misidentified [24, 31]. This is in line with observations in our laboratory. The vast majority of samples coming through our clinical microbiology laboratory are easily resolvable as species using standard methods. Nevertheless, the identification here of 35 potentially novel taxa, seven of which appear to have had a clinically relevant role, shows that there is still a wide range of undescribed bacterial organisms from clinical samples. Clinical microbiologists and infectious disease specialists should be aware of this spectrum and we encourage other laboratories to apply or to adapt our algorithm to improve the identification of difficult to identify isolates. A next important step within our NOVA study will be the correct taxonomical description of these isolates.

## Conclusions

To conclude, we developed an algorithm to characterize strains which are not identifiable by standard methods using WGS that allowed the identification of multiple, potentially novel taxa as well as difficult to identify strains. Public availability of corresponding genome sequences and detailed clinical information may help to expand the clinical and ecological understanding regarding these novel bacterial organisms.

### Electronic supplementary material

Below is the link to the electronic supplementary material.

**Supplementary Material 1:** Supplemental material is available online only. Supplemental Table S1.

## Data Availability

Genome data of 56 isolates of this study is accessible at NCBI under BioProject number PRJEB55530. Genome data of *Gulosibacter hominis* strains USB_NOVA_36, USB_NOVA_37, and USB_NOVA_38 are available under CAJGWQ000000000, CAJHCD000000000, and CAJHCF000000000, respectively [13]. The genomes of *Pseudoclavibacter triregionum* (USB_NOVA_49) [14] and *Cutibacterium modestum* (USB_NOVA_51) [15] are accessible under OU365335 and PRJEB41775, respectively. Scripts for calculating ANI-values between two genomes are deposited in github [https://github.com/schlaepferp/win_batch_ani].

## Abbreviations

WGS: Whole Genome Sequencing
