# Supplementary material for: Novel Organism Verification and Analysis (NOVA) study: identification of 35 clinical isolates representing potentially novel bacterial taxa using a pipeline based on whole genome sequencing
Source: BMC Microbiol. 2024 Jan 5;24:14. doi: 10.1186/s12866-023-03163-7 (PMC10768270; doi:10.1186/s12866-023-03163-7)
Supplement: Supplementary file 1 — Supplementary Material 1: Supplemental material is available online only. Supplemental Table S1. [file 12866_2023_3163_MOESM1_ESM.docx]

| **Supplementary material**  **Title:** Novel Organism Verification and Analysis (NOVA) study: identification of 35 clinical isolates representing potentially novel bacterial taxa using a pipeline based on whole genome sequencing  **Authors:** Veronika Muigg, Helena M.B. Seth-Smith, Kai-Manuel Adam, Maja Weisser, Vladimira Hinić, Annette Blaich, Tim Roloff, Ulrich Heininger, Hanna Schmid, Maurus Kohler, Lukas Graf, Dylan M. Winterflood, Pascal Schlaepfer, and Daniel Goldenberger  **Corresponding author:** Daniel Goldenberger, University Hospital Basel, Basel, Switzerland, e-mail address: daniel.goldenberger@usb.ch  **Table S1.** Detailed results of microbiological analyses from our bacterial strains (N=61) including references of taxonomic description | | | | | | | | |
| --- | --- | --- | --- | --- | --- | --- | --- | --- |
| **Sample ID** | **Type of Specimen** | **Collection date (MM.YYYY)** | **Culture result (amount)** | **Gram stain** | **Morphology** | **Maldi TOF MS result** | **Partial 16S rRNA gene sequencing result_percent identity_NCBI reference number_1^st^ hit** | **Partial 16S rRNA gene sequencing result_percent identity_NCBI reference number_2^nd^ hit** |
| USB_NOVA_12 | Biopsy bone toe | 11.2016 | ***Anaerococcus* sp.nov. (++)**  *Enterobacter cloacae*-group (+)  *Pseudomonas aeruginosa* (+)  *Enterococcus faecalis* (+)  *Bacteroides fragilis* (+)  *Porphyromonas species* (+) | pos | cocci | No result | *Anaerococcus prevotii* 97.35%_NR_074575.1 | *Anaerococcus* *prevotii* 96.79%_NR_041939.1 |
| USB_NOVA_36 / USB401352-2018 (Ref 13) | Swab outer ear channel | 04.2018 | ***Gulosibacter hominis**[1] (+++)**  *Corynebacterium aurimucosum* (+++)  *Corynebacterium amyculatum* (+++)  *Proteus mirabilis* (+++) | pos | coryne | No result | *Gulosibacter* *chungangensis* 97.48%_NR_109074.1 | *Gulosibacter* *bifidus* 96.65%_NR_024675.1 |
| USB_NOVA_43 | Biopsy bone symphysis | 03.2018 | ***Fenollaria massiliensis** [2]**  *Peptoniphilus coxii* | neg | rods | *Peptoniphilus gorbachii* score:1.78 / *Peptoniphilus tyrrelliae* score:1.81 | *Fenollaria* *massiliensis* 97.29%_NR_133038.1 | *Tissierella* *carlieri* 87.72%_NR_133038.1 |
| USB_NOVA_37 / USB401468-2018 (Ref 13) | Swab outer ear channel | 04.2018 | ***Gulosibacter hominis** [1](++)**  Skin flora (+) | pos | coryne | No result | Gulosibacter chungangensis 97.34%_NR_109074.1 | Gulosibacter bifidus 96.51%_NR_024675.1 |
| USB_NOVA_44 | Biopsy scrotum | 04.2018 | ***Mogibacterium kristiansenii** [3] (++)**  *Escherichia coli* (++)  *Enterococcus faecalis* (+)  *Proteus mirabilis* (+)  Anaerobic flora (++) | pos | rods | No result | *Mogibacterium* *vescum* 93.92%_NR_024712.1 | *Mogibacterium* *neglectum* 93.78%_NR_027203.1 |
| USB_NOVA_40 | Biopsy placenta fetal  site | 05.2018 | ***Corynebacterium pseudogenitalium** [4] (ae)**  *Corynebacterium amyculatum* (1 CFU)  *Parabacteroides distasonis* (ae)  *Finegoldia magna* (ae) | pos | coryne | No result | *Corynebacterium* *lipophiloflavum* 96.94%_NR_026370.1 | *Corynebacterium* *jeddahense* 96.93%_NR_144695.2 |
| USB_NOVA_07 | Biopsy jaw | 05.2018 | ***Schaalia* sp.nov. (+)**  *Streptococcus sanguinis* (+)  Non-pathogenic *Neisseria* species (+)  *Fusobacterium nucleatum* (+) | N.d. | N.d. | *Actinomyces georgiae* score:1.97 | *Schaalia* *georgiae* 98.30%_NR_026182.1 | *Schaalia* *meyeri* 96.73%_NR_029286.1 |
| USB_NOVA_20 | Abscess mamma | 07.2018 | ***Porphyromonas* sp.nov. (++)**  *Staphylococcus epidermidis* (ae)  *Prevotella bivia* (++) | neg | rods | No result | *Porphyromonas* *bennonis* 97.96%_NR_044491.1 | *Porphyromonas* *bennonis* 97.83%_NR_113228.1 |
| USB_NOVA_13 | Biopsy bone fibula | 10.2018 | ***Anaerococcus* sp.nov. (+)** | pos | cocci | No result | *Anaerococcus* *octavius* 95.86%_NR_026360.1 | *Anaerococcus* *nagyae* 94.66%_NR_146835.1 |
| USB_NOVA_42 | Swab finger nail | 10.2018 | ***“Kingella pumchi* “** [5] (+)**  *Staphylococcus epidermidis* (+++)  *Streptococcus pyogenes* (+++) | neg | coccoid rods | No result | *Kingella potus*_96.25%_NR_042240.1 | Neisseria bacilliformis_95.57%_NR_042978.1 |
| USB_NOVA_38 / USB404866-2018 | Swab outer ear channel | 11.2018 | ***Gulosibacter hominis** [1] (+++)**  *Skin flora* (++)  *Enterococcus faecalis* (++)  *Citrobacter freundii*-group (+++) | pos | rods | No result | *Gulosibacter* *chungangensis* 97.37%_NR_109074.1 | *Gulosibacter* *bifidus* 96.55%_NR_024675.1 |
| USB_NOVA_01 | Swab toe | 12.2018 | ***Corynebacterium* sp.nov. (+++)**  *Actinomyces neuii* (+++)  *Staphylococcus lugdunensis* (+++) | pos | rods | No result | *Corynebacterium* *camporealensis* 95.83%_NR_029326.1 | *Corynebacterium* *nasicanis* 95.96%_NR_145583.1 |
| USB_NOVA_21 | Swab outer ear channel | 12.2018 | ***Pusillimonas* sp.nov. (+++)**  *Staphylococcus epidermidis* (+)  *Corynebacterium amyculatum* (++)  *Corynebacterium striatum* (+++)  *Achromobacter* species (+++)  *Arthrographis kalrae* (+) | neg | rods | *Castellaniella defragrans* score:1.45 | *Advenella* *mimigardefordensis* 94.05%_NR_121716.1 | *Bordetella* *sputigena* 93.47%_NR_137343.1 |
| USB_NOVA_22 | Swab toe | 12.2018 | ***Dermabacter* sp.nov. (+++)**  *Pseudomonas aeruginosa* (++)  *Staphyloccocus lugdunensis* (++) | pos | rods | No result | *Dermabacter* *jinjuensis* 96.65%_NR_149775.1 | *Dermabacter* *vaginalis* 96.37%_NR_148832.1 |
| USB_NOVA_45 | Biopsy abscess plantar | 02.2019 | ***Anaerococcus degeneri** [6] (+)**  *Escherichia coli* (+)  *Streptococcus gordonii* (+)  Grampositive anaerobic rods (+) | pos | cocci | No result | *Anaerococcus* *degeneri* 97.78%_NR_146834.1 | *Anaerococcus* *murdochii* 97.23%_NR_115887.1 |
| USB_NOVA_46 | Biopsy abscess plantar | 02.2019 | ***Slackia exigua** [7] (+)**  *Escherichia coli* (+)  *Streptococcus gordonii* (+)  Grampositive anaerobic cocci (+) | pos | rods | *Slackia exigua* score:1.95 | *Slackia* *exigua* 98.59%_NR_024952.1 | *Slackia* *heliotrinireducens* 93.12%_NR_074439.1 |
| USB_NOVA_48 | Blood culture | 03.2019 | ***Devosia* *equisanguinis** [8]** | N.d. | N.d. | No result | *Devosia* *subaequoris* 98.10%_NR_042544.1 | *Devosia* *chinhatensis* 97.37%_NR_044214.1 |
| USB_NOVA_23 | Blood culture | 04.2019 | ***Helcococcus* sp.nov.**  *Murdochiella asaccharolytica* | pos | cocci | No result | *Helcococcus* *ovis* 89.50%_NR_027228.1 | *Helcococcus* *kunzii* 89.08%_NR_029237.1 |
| USB_NOVA_49 / 125703-19 | Blood culture | 06.2019 | ***Pseudoclavibacter triregionum** [9]** | pos | coryne | No result | *Pseudoclavibacter* *chungangensis* 95.67%_NR_116710.1 | *Chryseoglobus* *frigidaquae* 95.40%_NR_115999.1 |
| USB_NOVA_02 | Urine | 06.2019 | ***Corynebacterium* sp.nov. (10^5)**  *Lactobacillus* species (10^4) | pos | rods | No result | *Corynebacterium* *renale* 97.13%_NR_119174.1 | *Corynebacterium* *renale* 96.88%_NR_037069.1 |
| USB_NOVA_03 | Swab outer ear channel | 07.2019 | ***Corynebacterium* sp.nov. (++)**  Local flora (++)  *Pseudomonas aeruginosa* (+++) | pos | coryne | No result | *Corynebacterium* *renale* 96.81%_NR_037069.1 | *Corynebacterium* *renale* 97.68%_NR_119174.1 |
| USB_NOVA_24 | Swab limp | 10.2019 | ***Neisseria* sp.nov. (+++)**  *Streptococcus anginosus* (+++)  *Citrobacter freundii*-group (++)  *Staphylococcus aureus* (+) | neg | rods | No result | *Neisseria* *elongata* subsp. *nitroreducens* 96.70%_NR_104944.1 | *Neisseria* *elongata* subsp. *elongata* 94.74%_NR_025893.1 |
| USB_NOVA_25 | Biopsy hand | 10.2019 | ***Pseudomonas* sp.nov. (+)**  *Buttiauxella species* (1 CFU)  *Staphylococcus hominis* (1 CFU)  *Curtobacterium* species (+) | N.d. | N.d. | No result | *Pseudomonas* *benzenivorans* 98.10%_NR_116904.1 | *Pseudomonas* *taiwanensis* 97.69%_NR_116172.1 |
| USB_NOVA_26 | Biopsy hand | 10.2019 | ***Pantoea* sp.nov. (+)**  *Staphylococcus epidermidis* (2 CFU) | N.d. | N.d. | *Pantoea agglomerans* score:1.61 | *Pantoea* *stewartii* subsp. *indologenes* 97.98%_NR_104928.1 | *Pantoea* *stewartii* 97.84%_NR_044800.1 |
| USB_NOVA_04 | Blood culture | 11.2019 | ***Corynebacterium* sp.nov.** | N.d. | N.d. | *Corynebacterium* species score:1.49 | *Corynebacterium* *pilbarense* 96.96%_NR_116953.1 | *Corynebacterium* *ureicelerivorans* 96.46%_NR_042558.1 |
| USB_NOVA_50 | Swab lower leg | 11.2019 | ***Pseudomonas yangonensis** [10] (+++)**  *Staphylococcus epidermidis* (++) | N.d. | N.d. | *Pseudomonas mendocina* score:2.1 / *Pseudomonas oleovorans* score:1.7 | *Pseudomonas* *alcaliphila* 98.10%_NR_024734.1 | *Pseudomonas* *alcaliphila* 98.10%_NR_114072.1 |
| USB_NOVA_08 | Swab mouth | 10.2019 | ***Schaalia* sp.nov. (+)**  *Candida albicans* (++)  *Streptococcus theromphilus* (+)  *Enterobacter cloacae*-group (+++)  *Citrobacter freundii*-groupe (+++) | pos | rods | No result | *Schaalia* *odontolytica* 96.91%_NR_114395.1 | *Schaalia* meyeri 96.09%_NR_*029286*.1 |
| USB_NOVA_05 | Blood culture | 11.2019 | ***Corynebacterium* sp.nov.** | N.d. | N.d. | No result | *Corynebacterium* *pilbarense* 93.66%_NR_116953.1 | *Corynebacterium* *afermentans* subsp. *lipophilum* 93.65%_NR_044865.1 |
| USB_NOVA_47 | Biopsy bone heel | 11.2019 | ***Corynebacterium* *hindlerae** [11]**  *Staphylococcus aureus* | pos | rods | No result | *Corynebacterium* *halotolerans* 95.37%_NR_102500.2 | *Corynebacterium* *epidermidicanis* 95.34%_NR_108533.1 |
| USB_NOVA_09 | Biopsy submandibular | 01.2020 | ***Schaalia* sp.nov. (ae)**  Anaerobic flora (++) | pos | rods | No result | *Schaalia* *odontolytica* 95.10%_NR_041983.1 | *Shaalia* *odontolytica* 95.10%_NR_114395.1 |
| USB_NOVA_51 / 602588-20-USB | Sonicated fluid prosthetic hip | 03.2020 | ***Cutibacterium* *modestum** [12] (80 CFU/ml)**  *Staphylococcus capitis* (> 1000 CFU/ml) | pos | rods | No result | *Cutibacterium* *acnes* 97.59%_NR_113028.1 | *Cutibacterium* *acnes* 97.59%_NR_040847.1 |
| USB_NOVA_52 | Blood culture | 03.2020 | **Pseudoramibacter alactolyticus* [13]** | pos | rods | No result | *Pseudoramibacter* *alactolyticus* 98.23%_NR_112097.1 | *Eubacterium* *barkeri* 91.28%_NR_044661.2 |
| USB_NOVA_14 | Biopsy hand | 12.2014 | ***Clostridium* sp.nov. (ae)** | pos | rods | No result | *Clostridium paraputrificum* 94.83%_NR_113021.1 | *Clostridium algidicarnis*_94.56%_NR_041746.1 |
| USB_NOVA_27 | Swab maxilla | 05.2020 | ***Lancefieldella* sp.nov. (++)**  Anaerobic flora (++) | pos | rods | No result | *Lancefieldella* *rimae* 98.34%_NR_113038.1 | *Lancefieldella* *rimae* 98.34%_NR_036819.1 |
| USB_NOVA_28 | Swab sacral | 05.2020 | ***Rothia* sp. nov. (+)**  *Staphylococcus hemolyticus* (1 CFU)  *Staphylococcus hominis* (2 CFU)  *Finegoldia magna* (++)  *Prevotella buccalis* (+) | pos | cocci | No result | *Rothia* *amarae* 98.06%_NR_029045.1 | *Rothia* *terrae* 96.69%_NR_043968.1 |
| USB_NOVA_53 | Aspirate bile | 06.2020 | ***Enterococcus* *dongliensis** [14](+)**  *Streptococcus anginosus* (+) | pos | cocci | No result | *Enterococcus* *devriesei* 99.46%_ NR_042389.1 | *Enterococcus* *xiangfangensis* 99.64%_NR_133741.1 |
| USB_NOVA_06 | Urine | 06.2020 | ***Corynebacterium* sp.nov. (10^6)** | N.d. | N.d. | *Corynebacterium* lipophile group F1 score:1.93 | *Corynebacterium* *liangguodongii* 97.47%_NR_174247.1 | *Corynebacterium* *pilbarense* 97.47%_NR_116953.1 |
| USB_NOVA_15 | Blood culture | 06.2020 | ***Clostridium* sp.nov.** | pos | rods | No result | *Clostridium* *huakuii* 98.33%_NR_134006.1 | *Clostridium* *punense* 98.33%_NR_145903.1 |
| USB_NOVA_29 | Outer ear channel swab | 07.2020 | ***Pseudoclavibacter* sp.nov. (+++)**  Non-pathogen *Neisseria species* (+++)  *Staphylococcus hemolyticus* (1 CFU)  *Aspergillus fumigatus* (1 CFU)  *Neisseria mucosa* (+++)  *Enterococcus faecalis* (++) | pos | rods | No result | *Gulosibacter bifidus*_98.75%_NR_024675.1 | *Gulosibacter chungangensis*_97.21%_NR_109074.1 |
| USB_NOVA_18 | Biopsy abdomen | 07.2020 | ***Peptoniphilus* sp.nov. (+)**  Anaerobic flora (*+*)  *Corynebacterium amycolatum* (+)  *Staphylococcus epidermidis* (+)  *Proteus mirabilis* (1 CFU) | pos | cocci | No result | *Peptoniphilus* *coxii* 95.13%_ NR_117556.1 | *Peptoniphilus* *ivorii* 93.81%_NR_026359.1 |
| USB_NOVA_54 | Biopsy abdomen | 07.2020 | ***Prevotella brunnea** [15] (2 CFU)**  *Corynebacterium amycolatum* (+)  *Peptoniphilus* species (+) | neg | rods | No result | *Prevotella* *corporis* 94.90%_NR_113099.1 | *Prevotella* *corporis* 94.63%_NR_044627.1 |
| USB_NOVA_16 | Swab abdomen | 07.2020 | ***Desulfovibrio* sp.nov. (+)**  *Enterococcus faecium* (++) | neg | rods | No result | *Desulfovibrio* *desulfuricans* 95.72%_NR_104990.1 | *Desulfovibrio* *legallii* 95.21%_NR_108301.1 |
| USB_NOVA_10 | Biopsy lung | 09.2020 | ***Schaalia* sp.nov.** | pos | rods | No result | *Schaalia* *odontolytica* 96.73%_NR_041983.1 | *Schaalia* *odontolytica* 96.73%_NR_114395.1 |
| USB_NOVA_55 | Biopsy tibia | 09.2020 | ***Parvimonas* *parva** [16]**  *Corynebacterium coyleae* (ae)  *Peptoniphilus coxii* (+) | pos | cocci | *Parvimonas micra* score:1.66 | *Parvimonas* *micra* 97.53%_NR_114675.1 | *Parvimonas* *micra* 97.53%_NR_114338.1 |
| USB_NOVA_11 | Swab jaw | 09.2020 | ***Schaalia* sp.nov. (ae)**  *Rothia mucilaginosa* (+) | pos | cocci | No result | *Schaalia* *odontolytica* 97.26%_NR_041983.1 | *Shaalia* *odontolytica* 97.26%_NR_114395.1 |
| USB_NOVA_56 | Swab vaginal | 12.2020 | ***Kingella negevensis** [17] (++)**  Vaginal flora (+) | neg | rods | No result | *Kingella negevensis* 98.54%_NR_157664.1 | *Kingella kingae* 96.14%_NR_042976.1 |
| USB_NOVA_30 | Biopsy finger | 01.2021 | ***Tessaracoccus* sp.nov. (2 CFU)** | pos | cocci | No result | *Tessaracoccus defluvii* 93.86%_NR_146852.1 | *Tessaracoccus lapidicaptus* 93.73%_NR_134214.1 |
| USB_NOVA_17 | Blood culture | 02.2021 | ***Desulfovibrio* sp.nov.** | neg | rods | No result | *Desulfovibrio* *desulfuricans* 95.92%_NR_104990.1 | *Desulfovibrio* *legallii* 95.14%_NR_108301.1 |
| USB_NOVA_41 | Urine | 03.2021 | ***Corynebacterium* *pseudogenitalium** [4] (10^6)**  *Pseudomonas aeruginosa* (10^6) | pos | coryne | No result | *Corynebacterium* *liangguodongii* 94.92%_NR_174247.1 | *Corynebacterium* *jeddahense* 94.92%_NR_144695.2 |
| USB_NOVA_31 | Swab rectal | 04.2021 | *Citrobacter* sp.nov. | N.d. | N.d. | *Citrobacter* *amalonaticus* score:2.26 /*Citrobacter* *farmeri* score:2.23 | Citrobacter farmeri_98.11%_NR024861.1 | Salmonella enterica subsp. indica_98.11%_NR_044370.1 |
| USB_NOVA_32 | Aspirate pleura | 04.2021 | ***Paenibacillus* sp.nov. (ae)** | neg | rods | No result | *Paenibacillus* *cavernae* 92.98%_NR_148613.1 | *Paenibacillus* *turicensis* 91.42%_NR_114621.1 |
| USB_NOVA_57 | Urine | 04.2021 | ***“Corynebacterium* provencense”** [18] (10^5)**  Local flora (10^3) | pos | coryne | *Corynebacterium* *variabile* Score:1.39 | *Corynebacterium* *variabile* 98.61%_NR_025314.1 | *Corynebacterium* terpenotabidum 98.56%_NR_121699.2 |
| USB_NOVA_58 | Biopsie thumb | 05.2021 | ***Vandammella animalimorsus** [19]** | neg | rods | No result | *Corticibacter* *populi* 95.99%_NR_137376.1 | *Xenophilus* *azovorans* 95.21%_NR_025114.1 |
| USB_NOVA_39 | Biopsy foot | 12.2021 | ***Gulosibacter hominis** [1] (1CFU)**  *Staphylococcus epidermidis* (2 CFU)  *Enterococcus faecalis* (ae) | pos | coryne | No result | Gulosibacter chungangensis_97.23%_NR_109074.1 | Gulosibacter bifidus_96.4%_NR_024675.1 |
| USB_NOVA_59 | Swab rectal | 07.2021 | ***Saezia sanguinis** [20]** | neg | rods | No result | *Schlegelella* *thermodepolymerans* 92.10%_NR_025673.1 | *Azohydromonas* *ureilytica* 93.14%_NR_149204.1 |
| USB_NOVA_19 | Biopsy upper leg | 07.2021 | ***Peptoniphilus* sp.nov. (+)**  *Staphylococcus aureus* (+)  *Staphylococcus epidermidis* (+)  *Actinomyces funkei* (+)  *Cutibacterium avidum* (+)  *Porphyromonas* species (+) | pos | cocci | No result | *Peptoniphilus* *coxii* 95.92%_NR_117556.1 | *Peptoniphilus* *ivorii* 93.94%_NR_026359.1 |
| USB_NOVA_33 | Bronchial  secrete | 08.2021 | ***Ochrobactrum* (*Brucella*) sp.nov. (+)**  *Candida albicans* (+++)  Oral flora (+) | neg | rods | No result | *Brucella* *pseudointermedius* 97.37%_NR_043756.1 | *Brucella* *gallinifaecis* 96.22%_NR_025576.1 |
| USB_NOVA_34 | Swab pharynx | 10.2021 | ***Sneathia* sp.nov. (++)** | neg | rods | No result | *Sneathia* *sanguinegens* 94.71%_NR_118342.1 | *Sneathia* *sanguinegens* 94.58%_NR_025487.1 |
| USB_NOVA_35 | Blood culture | 01.2022 | ***Psychrobacter* sp.nov.**  *Corynebacterium coyleae* | neg | cocci | *Psychrobacter* *phenylpyruvicus* score:1.73 | *Psychrobacter* *submarinus* 97.83%_NR_025457.1 | *Psychrobacter* *pasteurii* 97.81%_NR_157988.1 |
| USB_NOVA_60 | Biopsy back | 01.2022 | ***„Corynebacterium* phoceense“** [21]** | pos | coryne | *Corynebacterium* species score:2.32 | *Corynebacterium* *lizhenjunii* 96.76%_MT044135.1 | *Corynebacterium* *argentoratense* 96.77%_NR_121753.2 |
| USB_NOVA_61 | Swab forehead | 01.2022 | ***Pantoea agglomerans** [22] (+++)**  *Pantoea* species (+++)  Skin flora (+) | N.d. | N.d. | No result | *Chimaeribacter* *arupi* 99.33%_MK530421.1 | *Chimaeribacter* *californicus* 99.06%_MK530419.1 |

Abbreviations. ID, identification; amount: +, detached; ++, moderate; +++, numerous; ae, after enrichment; CFU, colony forming units;

neg, negative; pos, positive; N.d., not done;

*already validly published; ** already published but not validly.

References

1. Vandamme P, Peeters C, Seth-Smith HMB, Graf L, Cnockaert M, Egli A, et al. Gulosibacter hominis sp. nov.: a novel human microbiome bacterium that may cause opportunistic infections. Antonie Van Leeuwenhoek. 2021;114(11):1841-54. <https://doi.org/10.1007/s10482-021-01644-1>.

2. Pagnier I, Croce O, Robert C, Raoult D, La Scola B. Non-contiguous finished genome sequence and description of Fenollaria massiliensis gen. nov., sp. nov., a new genus of anaerobic bacterium. Stand Genomic Sci. 2014;9(3):704-17. <https://doi.org/10.4056/sigs.3957647>.

3. Wylensek D, Hitch TCA, Riedel T, Afrizal A, Kumar N, Wortmann E, et al. A collection of bacterial isolates from the pig intestine reveals functional and taxonomic diversity. Nat Commun. 2020;11(1):6389. <https://doi.org/10.1038/s41467-020-19929-w>.

4. Jaen-Luchoro D, Al-Shaer S, Pineiro-Iglesias B, Gonzales-Siles L, Cardew S, Jensie-Markopolous S, et al. Corynebacterium genitalium sp. nov., nom. rev. and Corynebacterium pseudogenitalium sp. nov., nom. rev., two old species of the genus Corynebacterium described from clinical and environmental samples. Res Microbiol. 2023;174(1-2):103987. <https://doi.org/10.1016/j.resmic.2022.103987>.

5. Xiao M, Liu R, Du J, Liu R, Zhai L, Wang H, et al. Kingella pumchi sp. nov., an organism isolated from human vertebral puncture tissue. Antonie Van Leeuwenhoek. 2023;116(2):143-51. <https://doi.org/10.1007/s10482-022-01786-w>.

6. Veloo AC, Elgersma PE, van Winkelhoff AJ. Anaerococcus degenerii sp. nov., isolated from human clinical specimens. Anaerobe. 2015;33:71-5. <https://doi.org/10.1016/j.anaerobe.2015.02.002>.

7. Wade WG, Downes J, Dymock D, Hiom SJ, Weightman AJ, Dewhirst FE, et al. The family Coriobacteriaceae: reclassification of Eubacterium exiguum (Poco et al. 1996) and Peptostreptococcus heliotrinreducens (Lanigan 1976) as Slackia exigua gen. nov., comb. nov. and Slackia heliotrinireducens gen. nov., comb. nov., and Eubacterium lentum (Prevot 1938) as Eggerthella lenta gen. nov., comb. nov. Int J Syst Bacteriol. 1999;49 Pt 2:595-600. <https://doi.org/10.1099/00207713-49-2-595>.

8. Kampfer P, Busse HJ, Clermont D, Criscuolo A, Glaeser SP. Devosia equisanguinis sp. nov., isolated from horse blood. International journal of systematic and evolutionary microbiology. 2021;71(11). <https://doi.org/10.1099/ijsem.0.005090>.

9. Vandamme P, Peeters C, Seth-Smith HMB, Schmid H, Cnockaert M, Egli A, et al. Description of Pseudoclavibacter triregionum sp. nov. from human blood and Pseudoclavibacter albus comb. nov., and revised classification of the genus Pseudoclavibacter: proposal of Caespitibacter gen. nov., with Caespitibacter soli comb. nov. and Caespitibacter caeni comb. nov. Antonie Van Leeuwenhoek. 2022;115(4):461-72. <https://doi.org/10.1007/s10482-022-01712-0>.

10. Tohya M, Watanabe S, Teramoto K, Tada T, Kuwahara-Arai K, Mya S, et al. Pseudomonas yangonensis sp. nov., isolated from wound samples of patients in a hospital in Myanmar. International journal of systematic and evolutionary microbiology. 2020;70(6):3597-605. <https://doi.org/10.1099/ijsem.0.004181>.

11. Bernard KA, Burdz T, Pacheco AL, Wiebe D, Bernier AM. Corynebacterium hindlerae sp. nov., derived from a human granuloma, which forms black colonies and black halos on modified Tinsdale medium but is not closely related to Corynebacterium diphtheriae and related taxa. International journal of systematic and evolutionary microbiology. 2021;71(8). <https://doi.org/10.1099/ijsem.0.004919>.

12. Goldenberger D, Sogaard KK, Cuenod A, Seth-Smith H, de Menezes D, Vandamme P, et al. Cutibacterium modestum and Propionibacterium humerusii represent the same species that is commonly misidentified as Cutibacterium acnes. Antonie Van Leeuwenhoek. 2021;114(8):1315–20. [https://doi](https://urldefense.com/v3/__https://doi__;!!EDSXHx-qqdzzoNk!oy_OFK4Pxb0R2-SET8uYWL95F8quDl8NRblaxDPmadLtIr2Do5sMAdUpMEt-YQsuwy3owG035oJ0ypjON0FrBn1rZSvBU-M$).org/10.1007/s10482-021-01589-5.

13. Willems A, Collins MD. Phylogenetic relationships of the genera Acetobacterium and Eubacterium sensu stricto and reclassification of Eubacterium alactolyticum as Pseudoramibacter alactolyticus gen. nov., comb. nov. Int J Syst Bacteriol. 1996;46(4):1083-7. <https://doi.org/10.1099/00207713-46-4-1083>.

14. Li YQ, Gu CT. Enterococcus pingfangensis sp. nov., Enterococcus dongliensis sp. nov., Enterococcus hulanensis sp. nov., Enterococcus nangangensis sp. nov. and Enterococcus songbeiensis sp. nov., isolated from Chinese traditional pickle juice. International journal of systematic and evolutionary microbiology. 2019;69(10):3191-201. <https://doi.org/10.1099/ijsem.0.003608>.

15. Buhl M, Dunlap C, Marschal M. Prevotella brunnea sp. nov., isolated from a wound of a patient. International journal of systematic and evolutionary microbiology. 2019;69(12):3933-8. <https://doi.org/10.1099/ijsem.0.003715>.

16. Jansson MK, Hering S, Buhl MEJ. Parvimonas parva sp. nov., derived from a human genito-urinary lesion. International journal of systematic and evolutionary microbiology. 2021;71(12). <https://doi.org/10.1099/ijsem.0.005100>.

17. El Houmami N, Bakour S, Bzdrenga J, Rathored J, Seligmann H, Robert C, et al. Isolation and characterization of Kingella negevensis sp. nov., a novel Kingella species detected in a healthy paediatric population. International journal of systematic and evolutionary microbiology. 2017;67(7):2370-6. <https://doi.org/10.1099/ijsem.0.001957>.

18. Ndongo S, Andrieu C, Fournier PE, Lagier JC, Raoult D. 'Actinomyces provencensis' sp. nov., 'Corynebacterium bouchesdurhonense' sp. nov., 'Corynebacterium provencense' sp. nov. and 'Xanthomonas massiliensis' sp. nov., 4 new species isolated from fresh stools of obese French patients. New Microbes New Infect. 2017;18:24-7. <https://doi.org/10.1016/j.nmni.2017.01.013>.

19. Bernard KA, Pacheco AL, Burdz T, Wiebe D, Bernier AM. Assignment of provisionally named CDC group NO-1 strains derived from animal bite wounds and other clinical sources, to genera nova in the family Comamonadaceae: description of Vandammella animalimorsus gen. nov., sp. nov. and Franklinella schreckenbergeri gen. nov., sp. nov. International journal of systematic and evolutionary microbiology. 2022;72(2). <https://doi.org/10.1099/ijsem.0.005247>.

20. Medina-Pascual MJ, Monzon S, Villalon P, Cuesta I, Gonzalez-Romo F, Valdezate S. Saezia sanguinis gen. nov., sp. nov., a Betaproteobacteria member of order Burkholderiales, isolated from human blood. International journal of systematic and evolutionary microbiology. 2020;70(3):2016-25. <https://doi.org/10.1099/ijsem.0.004010>.

21. Cresci M, Ibrahima Lo C, Khelaifia S, Mouelhi D, Delerce J, Di Pinto F, et al. Corynebacterium phoceense sp. nov., strain MC1(T) a new bacterial species isolated from human urine. New Microbes New Infect. 2016;14:73-82. <https://doi.org/10.1016/j.nmni.2016.09.001>.

22. Gavini F MJ, Beji A, Mielcarek C, Izard D, Kersters K, De Ley J. . Transfer of Enterobacter agglomerans (Beijerinck 1888) Ewing and Fife 1972 to Pantoea gen. nov. as Pantoea agglomerans comb. nov. and description of Pantoea dispersa sp. nov. Int J Syst Bacteriol 1989;39:337-45.
